# Overexcited MaxiK and K_ATP_ channels underlie obstructive jaundice-induced vasoconstrictor hyporeactivity of arterial smooth muscle

**DOI:** 10.1038/srep39246

**Published:** 2016-12-21

**Authors:** Ya-wei Yuan, Long Wang, Zhan-ying Lu, Yue Long, Ying-fu Jiao, Qiang Xia, Da-xiang Wen, Wei-feng Yu

**Affiliations:** 1 Department of Anesthesiology, Renji Hospital affiliated to Shanghai Jiaotong University School of Medicine, Shanghai, China; 2 Department of Anesthesiology and Intensive Care, Eastern Hepatobiliary Surgery Hospital, Second Military Medical University, Shanghai, China; 3 Department of Physiology and Key Laboratory of Molecular Neurobiology, Ministry of Education, Second Military Medical University, PR China; 4 Department of Liver Surgery, Renji Hospital affiliated to Shanghai Jiaotong University School of Medicine, Shanghai, China

## Abstract

Substantial evidence has shown that obstructive jaundice can induce vascular hyporesponsiveness. The present study was designed to investigate mechanisms of MaxiK channel and K_ATP_ underlying cholestasis-induced vascular dysfunction. The isolated thoracic aorta was used to explore norepinephrine (NE)-induced contraction. The function of MaxiK and K_ATP_ channels were investigated using whole-cell patch clamp recording. Compared with Sham group, NE-induced vascular contraction was blunted after bile duct ligation (BDL), which could not be ameliorated significantly after endothelial denudation. Charybdotoxin and glibenclamide induced a more pronounced recovery from vascular hyporesponsiveness to NE in BDL group compared with Sham group. BDL significantly promoted the charybdotoxin sensitive MaxiK current and K_ATP_ current in isolated aortic smooth muscle cells. In addition, the expression of auxiliary subunits (MaxiK-β1 and SUR2B) rather pore-forming subunits (MaxiK-α and Kir6.1) was significantly up-regulated after BDL. These findings suggest that MaxiK and K_ATP_ channels play an important role in regulating vascular hyporesponsiveness in BDL rats.

Patients with cholestasis are vulnerable to hypotension, kidney dysfunction and other postoperative complications^1^,^2^,^3^,^4^, which could be insidious and may significantly increase perioperative mortality and morbidity^5^. The mortality rate in surgical patients with obstructive jaundice is reported to be 16–18%. The incidence of acute renal failure (ARF) in patients requiring surgical relief of cholestasis is approximately 8–10%, in whom the mortality is about 70–80%^6^.

Extensive clinical and laboratory investigations have heightened the awareness of clinicians about the relationship between perioperative management, especially adequate organ perfusion maintenance, and frequent complications of obstructive jaundice. Impaired vascular reactivity in cholestasis has been explored in numerous studies both *in vivo* and *in vitro*. Cattell and colleagues^3^ reported that dogs with chronic bile duct ligation (BDL) manifested blunted response to vasoactive agents. Other studies^2^ also found that cholestatic patients requiring biliary surgery were more vulnerable to a hypotensive crisis after haemorrhage.

The exact mechanism underlying the susceptibility of hypertension in patients with obstructive jaundice remains unclear. Using patch-clamp techniques, Dopico and co-workers^7^ found that bile acids reversibly activated large-conductance Ca^2+^-activated K^+^ (BK_Ca_, MaxiK, Slo1) channels in rabbit mesenteric arterial smooth muscle cells, and that administration of MaxiK channel blockers could eliminate vasodilation. Meanwhile, Lavoie and others^8^ reported that hydrophobic bile salts could decrease the gallbladder smooth muscle activity via excess opening of the ATP-sensitive K^+^ (K_ATP_) channel. The aim of present study was to explore hyporesponsiveness mechanisms of obstructive jaundice by focusing special attention on the role of vascular smooth muscle MaxiK and K_ATP_ channels in freshly isolated rat thoracic aorta.

## Results

Animals in Sham group did not show any alteration in general status under the experimental conditions. On day 7 after surgery, the mean weight of the rats in BDL group was significantly lower than that in Sham group. The serum concentrations of TBIL, DBIL, ALP, SGPT and SGOT in BDL group increased markedly as compared with those in Sham group (p < 0.01) (Table 1).

### Obstructive jaundice-induced vascular hyporesponsiveness is not ameliorated significantly after removing the endothelium

**Figure 1:**
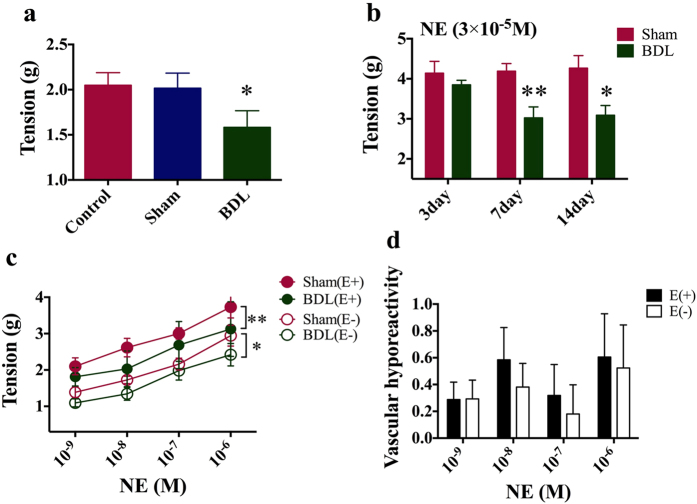
Obstructive jaundice-induced vascular hyporesponsiveness was not obviously ameliorated after removing the endothelium. (**a**) The basal vascular tone of thoracic aorta rings isolated from control, Sham and BDL groups. (**b**) The contractile response for arterial strips from rats with 3, 7 and14-day BDL or Sham to norepinephrine (3 × 10^−5^ M). (**c**) The contractile response for arterial strips from 7-day BDL rats to different doses of norepinephrine (10^−9^, 10^−8^, 10^−7^ and 10^−6^ M) with or without the endothelium. (**d**) Vascular hyporesponsiveness, as represented by subtraction of contractile tension between BDL and Sham groups at different doses of norepinephrine. *Represents p < 0.05, **p < 0.01, ***p < 0.001. E+ means strips with the endothelium, while E- means without the endothelium.

The basal vascular tone of the thoracic aorta rings isolated from the rats with obstructive jaundice was decreased markedly (Control: 2.05 ± 0.34 g; Sham: 1.95 ± 0.41 g; BDL: 1.6 ± 0.18 g, Fig. 1a). The contractile response for arterial strips to NE (3 × 10^−5^ M) in 7 and 14-day BDL rats was significantly blunted compared with that in Sham rats (3-day: Sham 4.14 ± 0.29 g vs. BDL 3.85 ± 0.11 g; 7-day: Sham 4.19 ± 0.19 g vs. BDL 3.03 ± 0.27 g; 14-day: Sham 4.27 ± 0.31 g vs. BDL 3.09 ± 0.24 g, Fig. 1b). There was also a significantly difference in endothelium-denuded arterial strips between Sham and BDL groups (10^–6^ M NE: Sham 2.94 ± 0.29 g vs. BDL 2.42 ± 0.75 g, Fig. 1c). Vascular hyporesponsiveness, as represented by subtraction of contractile tension between BDL and Sham groups, in normal arterial strips was comparable to that in endothelium-denuded arterial strips, indicating that the endothelium did not play a critical role in BDL-induce vascular hyporeactivity (10^−7^ M NE: E+ 0.32 ± 0.23 g vs. E- 0.18 ± 0.22 g, Fig. 1d).

### Vasoconstrictor hyporeactivity of BDL rats is blocked by inhibitors of MaxiK and K_ATP_ rather by K_v_ and K_ir_

**Figure 2:**
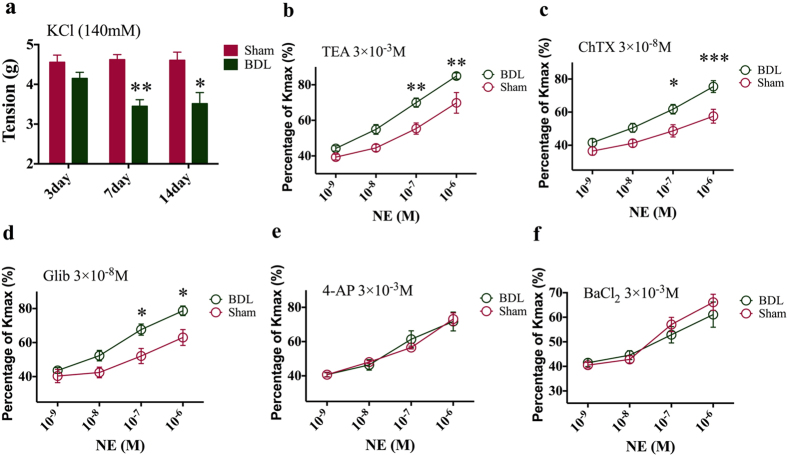
MaxiK and K_ATP_ in vascular smooth muscle cells played a critical role in vasoconstrictor hyporeactivity of BDL rats. (**a**) Vascular contractile response to KCl (140 mM) in 3, 7 and 14-day BDL or Sham rats. (**b**) Percentage of norepinephrine-induced contraction with TEA pretreatment in potassium-induced maximum contraction (K_max_) in 7-day Sham and BDL rats. (**c**) Percentage of norepinephrine-induced contraction with ChTX pretreatment in K_max_ in 7-day Sham and BDL rats. (**d**) Percentage of norepinephrine-induced contraction with Glib pretreatment in K_max_ in 7-day Sham and BDL rats. (**e**) Percentage of norepinephrine-induced contraction with 4-AP pretreatment in K_max_ in 7-day Sham and BDL rats. (**f**) Percentage of norepinephrine-induced contraction with BaCl_2_ pretreatment in K_max_ in 7-day Sham and BDL rats. *Represents p < 0.05, **p < 0.01, ***p < 0.001.

KCl (140 mM) induced a significant increase in vascular tone in Sham group as compared with BDL group (7-day: Sham 4.61 ± 0.50 g vs BDL 3.52 ± 0.68 g, Fig. 2a). To explore the role of potassium channels in vascular hyporeactivity, the percentage of NE induced contraction with potassium channel blockers pretreatment in potassium-induced maximum contraction (K_max_) was calculated (NE-induce contraction/K_max_) and the percentage represented the recovery from hyporesponsiveness induced by potassium channel blockers. After administration of TEA (tetraethylammounium, a non-selective potassium channel blocker, 3 × 10^−3^ M), the vasoconstrictor hyporeactivity of BDL rats was statistically reversed (10^−6^ M NE: Sham 69.8 ± 14.2% vs. BDL 84.9 ± 4.2%, Fig. 2b). Meanwhile, the contribution of MaxiK, K_ATP_, K_v_ and K_ir_ channels to regulate aortic tone was examined by calculating tension recovery generated by blocking the channel. ChTX (a specific MaxiK channel blocker) and Glib (a specific K_ATP_ channel blocker) produced a dramatic increase in NE induced-contractile response (ChTX: Sham 57.5 ± 10.2% vs. BDL 75.4 ± 8.7%; Glib: Sham 63.0 ± 11.4% vs. BDL 78.7 ± 6.9%, Fig. 2c,d), while the same effect was not observed after 4-AP (4-aminopyridine, a specific K_v_ channel blocker) and BaCl_2_ (barium chloride, a specific K_ir_ channel blocker) used (4-AP: Sham 73.2 ± 8.8% vs. BDL 71.8 ± 13.5%; BaCl_2_: Sham 66.1 ± 8.0% vs. BDL 61.1 ± 12.6%, Fig. 2e,f).

### MaxiK and K_ATP_ of aortic smooth muscle cells (ASMCs) isolated from the thoracic aorta of BDL rats are highly sensitized

**Figure 3:**
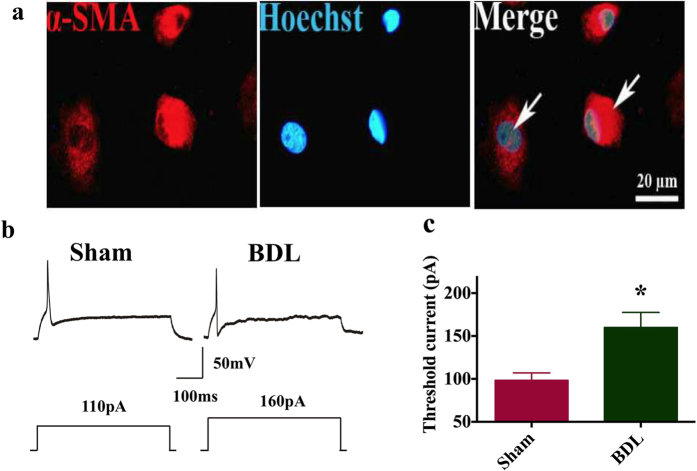
Action potential in ASMCs from Sham and BDL rats on postoperative day 7. (**a**) Purity of acute isolated aortic smooth muscle cells. (**b**) Representative responses during current injection in two ASMCs from Sham and BDL rats. (**c**) Histograms showed the mean threshold current intensities in Sham (n = 9) and BDL (n = 7) rats. BDL significantly enhanced the mean threshold intensities in BDL rats compared with that in Sham rats (P < 0.05).

ASMCs were isolated from the thoracic aorta using enzymatic digestion to obtain highly purified acute isolated ASMCs (Fig. 3a). Figure 3b illustrates the action potential generated by square-pulse stimulation in ASMCs of Sham and BDL rats. The mean threshold current intensity for evoking the action potential was 98.33 ± 8.72pA in Sham group (n = 6) vs. 160.3 ± 17.5pA in BDL group (n = 6). BDL significantly increased the threshold intensity as compared with Sham operation (Fig. 3c, p < 0.05).

**Figure 4:**
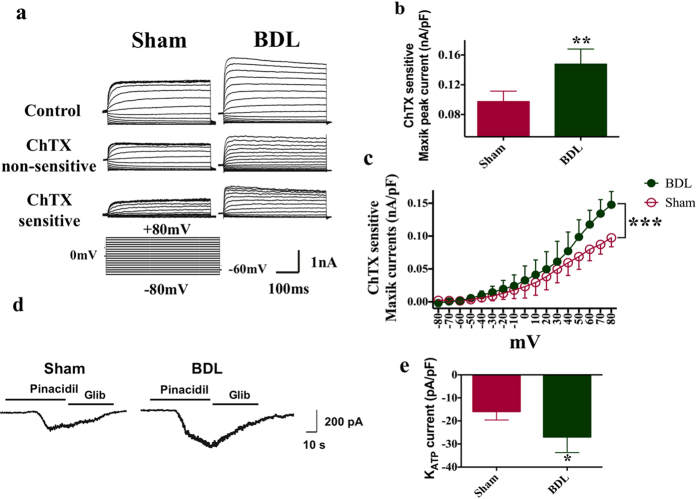
MaxiK and K_ATP_ currents in ASMCs from Sham and BDL rats on postoperative day 7. Whole-cell K^+^ currents in aortic myocytes measured by conventional whole-cell patch-clamp technique. (**a,b**) Typical recordings of MaxiK currents in ASMCs from Sham and BDL rats, respectively. In BDL (n = 11) groups, BDL BK_Ca_ peak currents were significantly larger at the voltage −60 mV, compared with those in Sham group (n = 10, ^*^P < 0.05). (**c**) The current-voltage relationship curves show differences in ASMCs between Sham (n = 10) and BDL (n = 11) rats. Compared with Sham group, ASMCs in BDL rats exhibited significantly larger MaxiK currents. (**d**) K_ATP_ currents of ASMCs from Sham and BDL rats. 10 μM pinacidil was administered to increase an inward current, and then it was reverted by glibenclamide to obtain pinacidil-induced K_ATP_ currents in ASMCs from both control and BDL models. (**e**) The magnitude of K_ATP_ current in ASMCs from BDL rats was significantly greater than that from Sham rats.

To evaluate the ensemble effect of MaxiK channels across the membrane of the entire cell, conventional whole-cell patch-clamp electrophysiology was used to measure the MaxiK currents during depolarizing voltage steps. As shown in Fig. 4a, under the same experimental conditions, the whole-cell MaxiK currents density in BDL was larger than that in Sham rats (n = 8). After ChTX (100 nM, 2 min) treatment, the whole-cell MaxiK currents were partly inhibited. It could be concluded that the ChTX-sensitive MaxiK current in BDL rats was larger than that in Sham rats (0.15 ± 0.01 nA/pF, n = 8 vs. 0.10 ± 0.01 nA/pF, n = 8, p < 0.01) (Fig. 4b). Figure 4c shows the mean current density vs. voltage plot. Aortic myocytes from BDL rats had an increased current density as compared with those from Sham rats.

Figure 4d showed the K_ATP_ currents of ASMCs from Sham and BDL rats. Whole cell recordings were carried out in a symmetrical 140 mM K^+^ solution to optimize the recordings, and the cells were held at a holding potential of −60 mV. Raising the extracellular K^+^ to 140 mM induced small KATP currents. 10 μM pinacidil was applied to increase an inward current in cells from Sham and BDL rats for enhancing the K_ATP_ currents (Fig. 4d), and glibenclamide (a K_ATP_ channel inhibitor) could revert it in both cell types. Then pinacidil-induced K_ATP_ currents in isolated ASMCs from both Sham and BDL models were obtained. The aortic smooth muscle cell resting membrane potential was −52.82 ± 1.19 mV and −51.35 ± 1.29 mV respectively in aortic smooth muscle cells from sham and BDL rats, and there was no significant difference. The membrane capacitance was 9.02 ± 0.45 pF in sham group and 8.90 ± 0.66 pF in BDL group. The magnitude of the K_ATP_ current in ASMCs from BDL rats was significantly greater than that from Sham rats (−26.94 ± 3.0αs. −15.91 ± 1.51pA/pF, Fig. 4e, p < 0.05).

### The increased expression of MaxiK-β1 and SUR2B rather than MaxiK-α and Kir6.1 is consistent with the high sensitivity of MaxiK and K_ATP_

**Figure 5:**
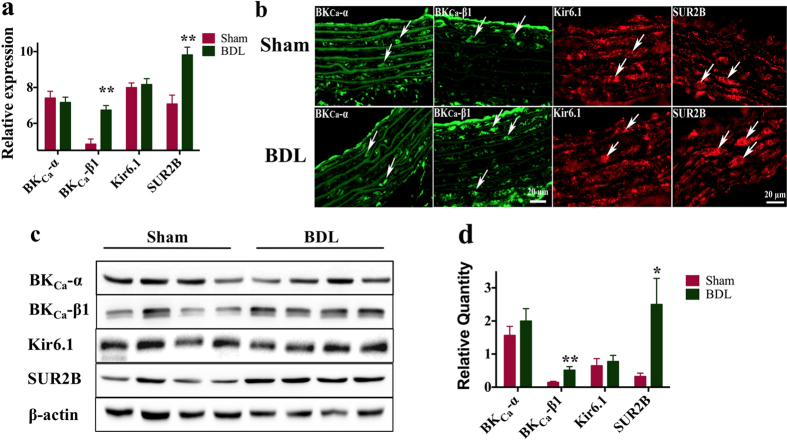
The effect of obstructive jaundice on K_ATP_ and MaxiK expression. (**a**) The expression of MaxiK-β1 subunit and K_ATP_ channel SUR2B subunit in aortic smooth muscle membranes was increased significantly as compared with that in Sham group, while obstructive jaundice had no effect on the expression of Kir6.1 and MaxiK-α. (**b**) Immunofluorescence showed that MaxiK-β1 and SUR2B were broadly expressed in ASMCs. (**c,d**) Expression of MaxiK-α, MaxiK-β1, Kir6.1 and SUR2B was confirmed by Western-blot. Results were expressed as mean ± SEM (n = 6). **P < 0.05.

It was determined whether the enhancement in K^+^ current was accompanied with any change in the expression of its subunit. qPCR analysis showed that BDL enhanced β1 transcripts by 1.39 fold, and SUR2B transcripts by 1.38 fold in the thoracic aorta tissue relative to Sham group (p < 0.01, Fig. 5a), whereas α and Kir6.1 did not show significant increase (p > 0.05, Fig. 5a). Immunofluorescence revealed that MaxiK-β1 subunit and K_ATP_-SUR2B subunit were mainly expressed in the ASMCs, and potentially up-regulated (Fig. 5b). Western blot analysis of MaxiK and K_ATP_ channel (n = 8) showed that the expression of β1 and SUR2B subunits was increased significantly after induction of obstructive jaundice, while the expression of α and Kir6.1 subunits remained unchanged significantly (Fig. 5c,d).

## Discussion

The principal finding of this work was that an unparallel alteration of MaxiK and K_ATP_ subunit expression underlay hypersensitive reaction of MaxiK and K_ATP_ channel activity and contribution to obstructive jaundice-induced vascular hyporeactivity, which is supported by the following findings: 1) ASMCs, instead of the endothelium, played a dominant role in obstructive jaundice-induced vascular hyporesponsiveness; 2) MaxiK and K_ATP_ channel inhibitors could significantly reverse vascular hyporesponsiveness of BDL; 3) MaxiK and K_ATP_ of ASMCs isolated from the thoracic aorta of BDL rats were highly sensitized; 4) The unparallel over-expressions of MaxiK-β1 and SUR2B, compared with MaxiK-α and Kir6.1, were observed in BDL group. Taken together, we firstly verified the role of MaxiK and K_ATP_ channels in vascular hyporesponsiveness of obstructive jaundice.

In our previous work^9^, we explored the vascular dysfunction in patients with obstructive jaundice, which is consistent with the finding of other studies^10^,^11^,^12^ that vascular function abnormalities in obstructive cholestasis could be attributed to blunted response to vasoactive agents. Other previous studies^13^,^14^ reported the blunted response of isolated femoral arteries from jaundiced dogs to NE, and this response disappeared after removal of the endothelium, which is inconsistent with our finding that there was still hyporesponsiveness to NE in endothelium-denuded vascular rings. This discrepancy may be due to different materials used, such as the vascular rings were prepared from the rat aorta in our study rather from the femoral artery in other studies.

Accumulating evidence shows that the activity of ion channels, especially the potassium channel, on ASMCs, play a pivotal role in affecting the contractile state of the peripheral arteries. Excess opening of the potassium channel in ASMC would cause membrane hyperpolarization of ASMC, finally resulting in vascular hyporeactivity^15^,^16^. Knowing that the compromised ability of an artery to constrict is likely to be caused by the defective function of the potassium channel in blood vessels and may be due to a change in unitary conductance, or open probability of the channels, and a change in expression number^17^, we detected the function and expression of MaxiK and K_ATP_ subunits using electrophysiological and molecular biology methods. It was found that an unparallel increase in MaxiK-β1 and K_ATP_-SUR2B subunits contributed to obstructive jaundice-related sensitization of these channels, which further damaged their function in regulating the vascular tone.

Arterial MaxiK channels consist of a pore-forming α-subunit in a tetrameric structure and co-assembled with a modulatory β-subunit in a 1:1 ratio^18^. The functional properties of the α-subunit are dramatically modified by the MaxiK-β1 subunit^18^,^19^,^20^, a critical molecular component in transferring calcium signals to the vasoregulated function^21^. Therefore, it is ritical to balance coupling of both subunits for maintaining arterial myocytes in a healthy state. β1-subunit deletion can blunt Ca^2+^ sensitivity of MaxiK channels and reduce coupling of Ca^2+^ sparks to MaxiK channel activation, thus increasing arterial tone and blood pressure^21^,^22^,^23^. Similarly, functional K_ATP_ channels in vascular smooth muscle consist of four Kir 6.1 pore-forming subunits and four SUR2B accessory subunits^24^. The critical role of the vascular K_ATP_ channel has been shown in mouse models of loss-of-function of SUR2 genes in the coronary circulation^25^. Notably, in hypertrophic animals, decreased K_ATP_ channel-induced vasorelaxation corresponding to decreased K_ATP_ current amplitude, is attributable to the down-regulation in expression of K_ATP_-SUR2 channel^26^. Transgenic over-expression of SUR2A in cardiac myocytes in SUR2 knockout mice significantly attenuated episodes of apparent coronary vasospasm^27^. In our study, a significant increase in MaxiK-β1 and SUR2B rather than MaxiK-α and Kir6.1 was observed in BDL group, compared with Sham group. Meanwhile ChTX-sensitive outward current (BK current) density and the magnitude of the K_ATP_ current were significantly increased in aortic myocytes of BDL rats with unparallel over-expression of MaxiK-β1 and SUR2B subunits. Based on the foregoing evidence, it is easy to speculate that unbalance in the molecular composition of MaxiK and K_ATP_ channels underlie alterations of channel functions, and further lead to vasoconstrictor hyporeactivity.

Bile acids may play a short-term role in regulating cardiovascular function not only by dose-dependent vasorelaxation^28^ but by interacting with MaxiK channel^29^. Also, the long-term effect of bile acids on vascular function is to modulate transcription of vasoactive molecules by targeting the transcription factor farnesyl X receptor (FXR)^30^,^31^. However, the role of bile acids and their receptors, especially FXR, in up-regulating MaxiK-β1 and SUR2B subunits remains unclear and needs to be further elucidated.

The present study has some limitations. First, our experiments were performed in rats, and we did not have clinical data to support our conclusion. Second, vascular hyporeactivity of BDL to NE has also been proved *in vivo*, which is manifested as lower mean arterial pressure. However, only the isolated thoracic aorta *in vitro* was used to explore the mechanisms of obstructive jaundice induced vascular hyporeactivity. In addition, vascular contractility was observed intuitively after ruling out other factors, such as blood volume and cardiac output. Finally, it would be desirable to down-regulate the β1 and SUR2B subunits of MaxiK and K_ATP_. More definitive causal relationship between β1 or SUR2B subunits and vascular hyporeactivity could be drawn if obstructive jaundice-induced vascular hyporesponsiveness could be obviously reversed after MaxiK-α and Kir6.1 subunit depletion.

In summary, our results indicate the impaired contractility of ASMCs in cholestasis could be attributable to the increased activity of the K_ATP_ channel and MaxiK channel. Such an effect is mediated by enhanced expression of β1 subunit and SUR2B. Our finding about cholestasis-induced vascular hyporeactivity mediated by alternation in MaxiK and K_ATP_ expression may shed new light on the mechanism of cardiovascular dysfunction in cholestasis and may help identify new therapeutic targets in this setting.

## Materials and Methods

### Animals

Adult male Sprague–Dawley (SD) rats weighing 220–250 g (Shanghai Laboratory Animal Center, Shanghai, China) were raised in a standard animal room with free access to water and chow, and under constant environmental conditions with a 12-h light-dark cycle. The rats were fasted for 12 h before surgery. The surgical procedures were approved by the Animal Care Committee of the Second Military Medical University (Shanghai, China) and performed in accordance with the guidelines from the Animal Care Committee of the said university.

### Chemicals

Unless otherwise stated, all reagents were purchased from Sigma-Aldrich (China).

### Experimental design and sample collection

Seventy-two rats were equally randomized to two groups. Obstructive jaundice was induced by BDL. Rats undergoing sham operation were allocated to Sham group, while normal rats served as the control group. Briefly, after anesthesia (2% pentobarbital sodium, intraperitoneal) and median laparotomy, the common bile duct was segregated and resected between a proximal and distal ligature. Bile ducts of rats in Sham group were similarly operated but neither resection nor ligation was carried out. On day 7 after surgery, the rats were sacrificed and the thoracic aorta was isolated for further investigation. Serum samples were stored at −80 °C for biochemical analysis.

### Thoracic aorta isolation and vascular reactivity

Twenty-four rats were adopted for study of vascular contractility and electrophysiology. The thoracic aorta was isolated quickly and placed in cold Krebs solution containing (mM): 131.5 NaCl, 2.5 CaCl_2_, 1.2 MgCl_2_, 5 KCl, 1.2 NaH_2_PO_4_, 13.5 NaHCO_3_, 11.2 glucose (pH 7.4, 37 °C), aerated with 95%O_2_ and 5%CO_2_. The aorta segments were cut into rings (3 mm long) and mounted on two stainless-steel wires passing through the vessel lumen. A stainless-steel rod was used to remove the endothelium by gently rubbing the luminal surface. Removal of the endothelium was verified by the lack of response to 10 μM acetylcholine. Arterial isometric tension recording was carried out with a MAP2000 isometric force transducer (Alcott Biotech Co., Ltd., Shanghai, China) connected to a computer. Each vessel device was placed in an individual 5 ml tissue bath.

Contractile response was evaluated by measuring the maximal peak height and expressed as the percentage of maximal tension achieved in response to 140 mM K^+^ (K_max_). Dose-response curves for NE (doses from 10^−9^ to 10^−6^ M) were obtained in aortic rings in a cumulative manner. To explore the role of MaxiK and K_ATP_ channels in vascular tension, the contractility was quantitated after administration of 3 × 10^−3^ M TEA (a non-selective potassium channel blocker), 3 × 10^−8^ M charybdotoxin (ChTX, a potent MaxiK channel blocker), 3 × 10^−8^ M glibenclamide (K_ATP_ channel blocker) and 3 × 10^−3^ M 4-AP (a potent Kv channel blocker) and BaCl_2_ (a potent Kir channel blocker).

### Western blot of MaxiK and K_ATP_

Thoracic aortas from different groups were collected and stored at −80 °C. The tissues were homogenized in ice-cold lysis buffer. Protein concentrations were measured according to a modified Bradford assay. Aliquots of proteins (40 μg) were separated by Mini-PROTEAN TGX Gel (Bio-Rad, USA) and transferred to the nitrocellulose filter by electroblotting. The transferred membrane was incubated with antibodies raised against MaxiK-α (1:1000; Alomone Labs, Israel), MaxiK-β1(1:1000; Alomone Labs), Kir 6.1 (1:500; Santa Cruz, USA), and SUR2B (1:500; Santa Cruz, USA) at 4 °C overnight, and then with a secondary antibody (anti-rabbit/anti-goat IgG-HRP, 1:3000, Cell Signaling Technology) for 2 h at room temperature. Protein concentrations were detected by chemiluminescence (ECL Plus Reagent kit, Bio-Rad) with Image Quant LAS 4000 (General Electric Company, GE, USA).

### Real-Time PCR of MaxiK and K_ATP_

Total RNA was extracted from the rat aorta with a RNAiso Plus kit (Takara) according to the manufacturer’s instruction. cDNA was reverse-transcribed from 2 μl total RNA in a 10 μl reaction. Amplification was performed in ABI 7900 HT Real-Time PCR System in a final volume of 20 μl. The primer sequences used in our experiment were list in Table 2.

### Immunofluorescence of MaxiK and K_ATP_

Aortic rings were harvested and fixed in 4% paraformaldehyde for 6 h, and then transferred into 20% sucrose, followed by 30% sucrose until they sank to the bottom. Frozen transverse sections through the aortic rings (10 μm thickness) were collected. The preparations were pre-incubated with blocking solution for 30 min, and then incubated with MaxiK-β1 (1:400, Alomone Labs), SUR2B (1:200, Santa Cruz) antibody at 4 °C overnight, and with Cy3-conjugated donkey anti-rabbit IgG (CST, 1:200) against MaxiK-β1 antibodies and FITC-conjugated donkey anti-goat IgG (CST, 1:200) against SUR2B antibodies for 4 h at room temperature. All the incubations and reactions were separated by 10-min PBS washing × 3. Immunofluorescence of ASMCs was similar to the above. The stained sections were observed with an epifluorescence microscope (Leica DMRE; Leica Microsystems, Rueil-Malmaison, France), and images were photographed through a Leica camera.

### ASMCs isolation and electrophysiological recording

ASMCs were isolated from the rat thoracic aorta in BDL or Sham groups by enzymatic digestion as described by Capponi and colleagues^32^. Growth of passage 1–2 ASMCs was arrested for 24 h in serum-free Dulbecco’s modified Eagle’s medium before electrophysiological experiments. Single cells were released using a fire-polished pipette and allowed to adhere to the bottom of a recording chamber (0.5 ml). All operations were performed at room temperature (20–25 °C).

Whole cell patch clamp recording was carried out with an Axopatch 700B amplifier (Axon Instruments, USA). The potential of the membrane was clamped at −60 mV, signals were filtered at 2 kHz (frequency 3 dB, Bessel filter, 80 dB/decade), subsequently digitized at 10–50 kHz (Digidata 1440 A interface, Axon Instruments). The resistance of patch electrodes was 4–5 MΩ. The conventional whole cell patch clamp technique was used to record the action potentials under current-clamp recording. The bath solution contained (in mM): 134 NaCl, 6 KCl, 1 MgCl_2_, 1.8 CaCl_2_, 10 glucoses and 10 HEPES (pH 7.4). The pipette solution contained the following (in mM): 107 KCl, 1.0 MgCl_2_, 1.9 CaCl_2_, 10 HEPES, 5 EGTA, 25 KOH, 0.1 Na_2_ATP, 0.1 NaADP and 0.1 LiGTP (pH 7.2 adjusted with KOH, free Ca^2+^~100 nM). The action potentials were recorded under current-clamp recording. During a 400 ms injection of a positive current (ranging from −40 to +350pA in 10pA increments), a single action potential could be evoked.

Transient outward BK_Ca_ currents were measured as described by Li and co-workers^33^. A pre-pulse (0 mV, 100 ms) was followed by test pulses (400 ms) from −80 to +80 mV in 10 mV increments. Test solutions bathing the cytoplasmic face of the patch membrane contained (in mM): 145 N-methyl-D-glucamine (NMDG), 3 KCl, 0.6 MgCl_2_•6H_2_O, 2.5 CaCl_2_•2H_2_O, 10 HEPES, 10 glucose (pH adjusted to 7.4 with Tris-base and 300 mOsM). ChTX (100 nM) was added to extracellular solutions to get the ChTX sensitive BK_Ca_ currents (The control currents subtracted the ChTX non-sensitive currents).

Freshly isolated VSMCs were obtained to record K_ATP_ currents at room temperature. With references to Koide and others^34^, K_ATP_ currents were recorded at −60 mV. After a stable baseline was obtained, the extracellular solution was changed to 140 mM K^+^ solution, and the “140 mM K^+^” bath solution was made by iso-osmotic replacement of NaCl with KCl. Pinacidil (10 μM) and glibenclamide (10 μM) were routinely added to extracellular solutions, pinacidil was used to increase an inward current, otherwise glibenclamide was used to inhibit the inward current, then the glibenclamide sensitive K_ATP_ currents were recorded.

### Statistical analysis

Quantitative data are presented as mean ± SD (SEM). Statistical analysis was performed using SPSS (version 15, SPSS Inc., Chicago, USA) software. The basal vascular tension between the control, Sham and BDL rats were analyzed using one-way ANOVA if significant by Dunnett’s multiple comparison tests. Then, a two-way repeated measures ANOVA was used to compare the response to different doses of NE between BDL and Sham groups, with again Holm-Bonferroni post hoc tests if a significant difference emerged. Independent-sample t test was used to assess differences between Sham and BDL groups. *P < 0.05 was considered statistically significant. The investigator who undertook statistical analysis did not know which rats belong to the BDL group, or the Sham group.

## Additional Information

**How to cite this article:** Yuan, Y.-w. *et al*. Overexcited MaxiK and K_ATP_ channels underlie obstructive jaundice-induced vasoconstrictor hyporeactivity of arterial smooth muscle. *Sci. Rep.* **6**, 39246; doi: 10.1038/srep39246 (2016).

**Publisher's note:** Springer Nature remains neutral with regard to jurisdictional claims in published maps and institutional affiliations.

## Figures and Tables

**Table 1:** Serum TBIL, DBIL, ALT, AST, SGOT and SGPT levels from 7-day BDL rats.

	Body weight (g)	TBIL (μM)	DBIL (μM)	ALP (IU/L)	SGOT (IU/L)	SGPT (IU/L)
Sham group	221.4 ± 20.3	1.2 ± 0.5	0.8 ± 0.4	37.1 ± 6.2	77 ± 11	43 ± 8
BDL group	201.7 ± 21.1^*^	126.8 ± 24.0^*^	114.6 ± 21.5^*^	337.5 ± 77.1^*^	473 ± 52^*^	218 ± 27^*^

Data are the mean ± SD (n = 6). ^*^p < 0.01 compared with Sham group.

ALP, alkaline phosphatase; TBIL, total bilirubin; DBIL, direct bilirubin; SGOT, serum glutamic oxaloacetic transaminase; SGPT, serum glutamic pyruvic transaminase.

**Table 2:** The primer sequences used in our experiment.

Genes	Primer sequence	
ABCC9	F:CACCTGGACAACTACGAGCA	R:ATCTCACCAAGGATGGCAAG
Kcnma1	F:TGTGGGCTCCATCGAGTA	R:TCCTTGTCCTGAAGCGAAGT
ACTB	F:TTGCTGACAGGATGCAGAAG	R:CAGTGAGGCCAGGATAGAGC
KCNJ8	F:GCCATCACGGTTTTGATTCT	R:GGTTTTCTTGACCACCTGGA
Kcnmb1	F:TCTCTTCTGCACAGCAGCAT	R:TGTGACTGGCAGTTCCTTTG
